# Gender-Based Differences by Age Range in Patients Hospitalized with COVID-19: A Spanish Observational Cohort Study

**DOI:** 10.3390/jcm10050899

**Published:** 2021-02-25

**Authors:** Claudia Josa-Laorden, Anxela Crestelo-Vieitez, María del Mar García Andreu, Manuel Rubio-Rivas, Marcos Sánchez, Neera Toledo Samaniego, Francisco Arnalich Fernández, Rosario Iguaran Bermudez, Eva Ma Fonseca Aizpuru, Juan Antonio Vargas Núñez, Paula Maria Pesqueira Fontan, Jorge Serrano Ballesteros, Santiago Jesús Freire Castro, Melani Pestaña Fernández, Alba Viana García, Victoria Nuñez Rodriguez, Vicente Giner-Galvañ, Francisco Javier Carrasco Sánchez, Almudena Hernández Milián, Marta Cobos-Siles, Jose Javier Napal Lecumberri, Virginia Herrero García, Maria de los Reyes Pascual Pérez, Jesús Millán Núñez-Cortés, José Manuel Casas Rojo, on behalf of the SEMI-COVID-19 Network

**Affiliations:** 1 Internal Medicine Department, Royo Villanova Hospital, Avenida San Gregorio 30, 50015 Zaragoza, Spain; mariadelmargarciaandreu@gmail.com; 2 Aragon Institute of Health Research, IIS-Aragon, 50009 Zaragoza, Spain; 3 Internal Medicine Department, Bellvitge University Hospital-IDIBELL, L’Hospitalet de Llobregat, 08901 Barcelona, Spain; mrubio@bellvitgehospital.cat; 4 Internal Medicine Department, 12 de Octubre University Hospital, 28041 Madrid, Spain; marcossanferab@gmail.com; 5 Internal Medicine Department, Gregorio Marañon University Hospital, 28007 Madrid, Spain; neera.toledo@salud.madrid.org (N.T.S.); jesus.millan@salud.madrid.org (J.M.N.-C.); 6 Internal Medicine Department, La Paz University Hospital, 28046 Madrid, Spain; farnalich@salud.madrid.org; 7 Internal Medicine Department, San Carlos Clinical Hospital, 28040 Madrid, Spain; draiguaran@gmail.com; 8 Internal Medicine Department, Cabueñes Hospital, 33394 Gijón, Spain; evamfonseca@yahoo.es; 9 Internal Medicine Department, Puerta de Hierro University Hospital, 28222 Majadahonda, Spain; juanantonio.vargas@uam.es; 10 Internal Medicine Department, Santiago Clinical Hospital, 15706 Santiago de Compostela, Spain; paulapesqueira@hotmail.com; 11 Internal Medicine Department, La Princesa University Hospital, 28006 Madrid, Spain; jserrano645@gmail.com; 12 Internal Medicine Department, A Coruña University Hospital, 15006 A Coruna, Spain; santiago.freire.castro@sergas.es; 13 Internal Medicine Department, Moisès Broggi Hospital, 08970 Sant Joan Despí, Spain; Melani.Pestana@sanitatintegral.org; 14 Internal Medicine Department, Dr. Peset University Hospital, 45017 Valencia, Spain; alvigar83@gmail.com; 15 Internal Medicine Department, Costa del Sol Hospital, 29603 Málaga, Spain; mijas29@hotmail.com; 16 General Internal Medicine Department, San Juan de Alicante University Hospital, 03550 Alicante, Spain; ginervicgal@gmail.com; 17 Department of Clinical Medicine, Faculty of Medicine, Miguel Hernández University, 03202 Alicante, Spain; 18 Internal Medicine Department, Juan Ramón Jiménez Hospital, 21005 Huelva, Spain; fjcarrascos@icloud.com; 19 Internal Medicine Department, Son Llàtzer University Hospital, 07120 Palma de Mallorca, Spain; ahernandez4@hsll.es; 20 Internal Medicine Department, Río Hortega University Hospital, Regional Health Management of Castilla y Leon (SACYL), 47012 Valladolid, Spain; mcoboss@saludcastillayleon.es; 21 Internal Medicine Department, Marqués de Valdecilla University Hospital, 39008 Santander, Spain; josejavier.napal@scsalud.es; 22 Internal Medicine Department, Doctor José Molina Orosa Hospital, 25005 Arrecife, Spain; virgyherrero@hotmail.com; 23 Internal Medicine Department, Elda University General Hospital, 03600 Alicante, Spain; cperezb@coma.es; 24 Internal Medicine Department, Infanta Cristina University Hospital, 28981 Madrid, Spain; jm.casas@gmail.com

**Keywords:** SARS-CoV-2, coronavirus, COVID-19, Spain, gender differences

## Abstract

There is some evidence that male gender could have a negative impact on the prognosis and severity of severe acute respiratory syndrome coronavirus 2 (SARS-CoV-2) infection. The aim of the present study was to compare the characteristics of coronavirus disease 2019 (COVID-19) between hospitalized men and women with confirmed SARS-CoV-2 infection. This multicenter, retrospective, observational study is based on the SEMI-COVID-19 Registry. We analyzed the differences between men and women for a wide variety of demographic, clinical, and treatment variables, and the sex distribution of the reported COVID-19 deaths, as well as intensive care unit (ICU) admission by age subgroups. This work analyzed 12,063 patients (56.8% men). The women in our study were older than the men, on average (67.9 vs. 65.7 years; *p* < 001). Bilateral condensation was more frequent among men than women (31.8% vs. 29.9%; *p* = 0.007). The men needed non-invasive and invasive mechanical ventilation more frequently (5.6% vs. 3.6%, *p* < 0.001, and 7.9% vs. 4.8%, *p* < 0.001, respectively). The most prevalent complication was acute respiratory distress syndrome, with severe cases in 19.9% of men (*p* < 0.001). In men, intensive care unit admission was more frequent (10% vs. 6.1%; *p* < 0.001) and the mortality rate was higher (23.1% vs. 18.9%; *p* < 0.001). Regarding mortality, the differences by gender were statistically significant in the age groups from 55 years to 89 years of age. A multivariate analysis showed that female sex was significantly and independently associated with a lower risk of mortality in our study. Male sex appears to be related to worse progress in COVID-19 patients and is an independent prognostic factor for mortality. In order to fully understand its prognostic impact, other factors associated with sex must be considered.

## 1. Introduction

Since the first cases of coronavirus disease 2019 (COVID-19), caused by severe acute respiratory syndrome coronavirus 2 (SARS-CoV-2), were described in China in December 2019, this virus has spread worldwide. As of January 2021, there have been more than 94 million confirmed infections, and over 2 million deaths worldwide. It has a 2.2% fatality rate [1,2,3].

Over 29.7 million infections have been recorded in Europe. In Spain, the first confirmed SARS-CoV-2 infection was reported on 31 January 2020, and since then, Spain has been one of the countries most affected by this pandemic, with 2,252,164 confirmed cases and 53,314 deaths as of 16 January 2021 [3,4].

The initial reports from China pointed to a difference in the number of cases detected and the fatality rate between the sexes. However, few reports have addressed this disparity in COVID-19 incidence and disease course according to sex; most of the studies that have been published so far indicate that male gender is linked to more serious forms of the disease, but more thorough investigation is necessary. As the disease has spread across multiple countries, the Global Health 50/50, an independent research initiative for gender equality, presented an overview of sex-disaggregated data from countries worldwide. Though a similar numbers of cases have been recorded in women and men, the fatality rate is higher among men; in January 2021, the number of COVID-19 deaths worldwide was divided into approximately 777,000 men, 578,000 women, and 633,000 deaths for which the sex was unknown. Nevertheless, sex-disaggregated data are still not provided by all countries, and the number of cases and the case fatality rate vary significantly by region [2,5,6].

In Spain, the Spanish Society of Internal Medicine (SEMI, for its initials in Spanish) has sponsored the SEMI-COVID-19 Network. The SEMI-COVID-19 Registry involved gathering detailed information on a large cohort of hospitalized patients. In this work, statistical analyses were carried out on data on baseline characteristics, comorbidities, clinical presentation, laboratory and radiological data, treatments received, clinical progress, complications, the need for ICU admission, and in-hospital mortality [7].

The aim of this study was to analyze the differences in the clinical characteristics, progress, and mortality rate of patients with COVID-19 according to sex. Understanding sex-based differences in this disease is essential for the expansion of the knowledge of the etiopathogenesis and epidemiology of the virus, as well as to more precisely analyse the social and economic impact that COVID-19 has on both patients and communities [1,8].

## 2. Materials and Methods

### 2.1. Study Design

This work is a multicenter, observational, cohort study of patients hospitalized due to COVID-19 in Spain. The Spanish Society of Internal Medicine is the sponsor of the nationwide SEMI-COVID-19 Registry, which retrospectively collects patient data. The specific characteristics of the registry have been described in previous works [7].

### 2.2. Population and Inclusion Criteria

The SEMI-COVID-19 Registry included data on hospitalized patients over 18 years of age with microbiologically-confirmed SARS-CoV-2 infection. All of the patients who were discharged or deceased after hospital admission with confirmed SARS-CoV-2 infection were candidates for inclusion. The infection was confirmed by a polymerase chain reaction (PCR) test of a nasopharyngeal, sputum, or bronchoalveolar lavage sample, or a positive result on a serological test with compatible symptoms.

In this work, the data on all of the patients in the registry were analyzed in order to examine the differences between men and women. The inclusion criteria were: (a) an age of 18 years or older; (b) a confirmed diagnosis of COVID-19; (c) a first hospital admission to a Spanish hospital participating in the study, and (d) a hospital discharge or a hospital death. The exclusion criteria were patients with incomplete data, and those who did not provide consent.

The data analyzed included baseline characteristics, comorbidities, clinical and radiological presentation, laboratory data, treatment, complications during hospitalization, and in-hospital mortality.

This record began collecting patients in March 2020, and we are currently still collecting data from hospitalized patients. The data in our study correspond to the first wave of patients hospitalized by COVID from March 2020 until July 2020.

### 2.3. Ethical Aspects

The SEMI-COVID-19 Registry and the studies related to it were approved by the Provincial Research Ethics Committee of Málaga (Spain). All of the included patients or their legal representatives gave their informed consent. In cases in which there were biological safety concerns, or in which the patient had already been discharged, consent was obtained verbally and noted on the patient’s medical record.

### 2.4. Data Analysis

The patients were divided into two groups: a male group and a female group.

The normality of the continuous variable distributions was verified using the Kolmogorov–Smirnov test. Data that follow a normal distribution are shown as means and standard deviations (SD), whereas data that follow a non-normal distribution are shown as medians and interquartile ranges (IQR). The comparisons between the groups for the normally distributed continuous variables were made using Student’s t-test. In cases in which the variables did not follow a normal distribution, the Mann-Whitney U test was used.

The categorical variables are expressed as absolute frequencies (*n*) and percentages (%). In order to compare the categorical variables, we used the chi-square test or Fisher’s exact test. The relationship between the variables and mortality was calculated using a multivariate logistic regression model which included the variables of gender, age, dependency, comorbidity, fever, dyspnea, confusion, tachypnea, severe acute respiratory distress syndrome [ARDS], radiological affectation, and radiological worsening at 7 days.

Statistical significance was established as *p* < 0.05. The statistical analyses were performed using SPSS version 25.0.

## 3. Results

**Figure 1:**
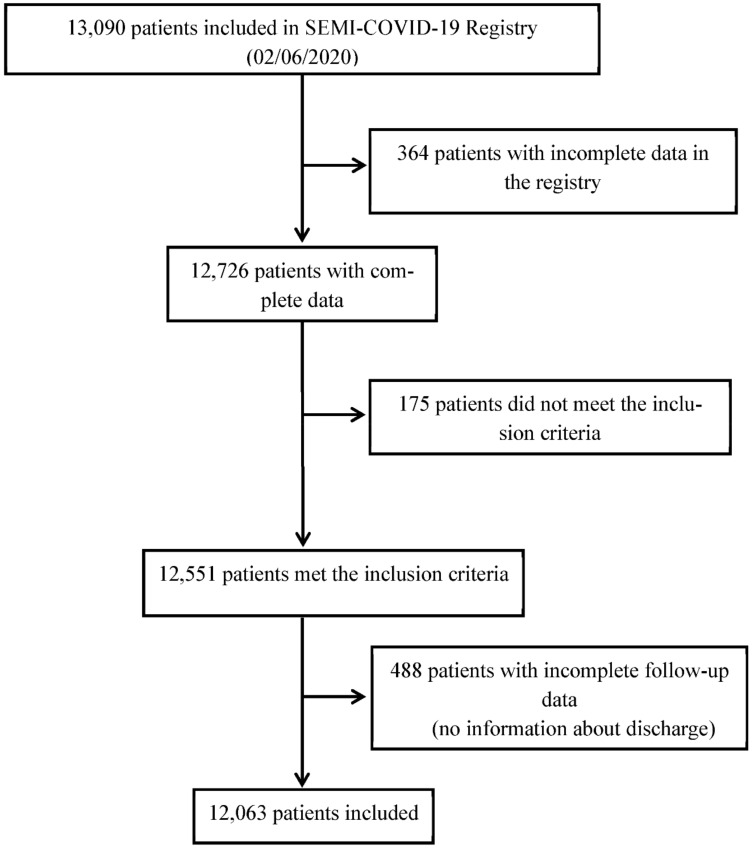
Patient inclusion flowchart.

A total of 13,090 patients were included in the SEMI-COVID-19 Registry as of 2 June 2020. Of them, this work analyzed data on 12,063 patients, of which 6853 were men (56.8%). Figure 1 shows the patient inclusion flowchart.

### 3.1. General Data at Baseline

In our study population, the women were older than the men, with a mean age of 67.9 vs. 65.7 years (*p* < 0.001). The notable findings on the baseline characteristics and comorbidities include a higher smoking rate observed among men (6.9% vs. 3.3%; *p* < 0.001) and a higher percentage of women with obesity (22.3% vs. 20.5%; *p* = 0.022). Severe dependency was more frequent in women (10.1% vs. 5.1%; *p* < 0.001). All of the data on the baseline characteristics and comorbidities are shown in Table 1.

### 3.2. Symptoms upon Admission

With regard to the clinical presentation of COVID-19, symptoms such as a cough, fever > 38 °C, dyspnea, tachypnea and oxygen saturation < 92% were more frequent among men, whereas milder symptoms such as odynophagia, ageusia, anosmia, arthralgia, headache, and abdominal symptoms were more frequent among women.

On the chest X-rays, bilateral involvement was more common than unilateral involvement. Bilateral condensation and bilateral interstitial infiltrates were significantly more frequent in men (31.8% vs. 29.9%; *p* = 0.007 and 53.7% vs. 48.6%; *p* < 0.001, respectively). Table 2 describes the clinical presentation of the COVID-19 disease, and the main differences observed between the groups.

### 3.3. Treatments

The data regarding the treatment received were also analyzed. Systemic steroids were widely used, with higher frequency among men, a finding that was statistically significant (38.7% vs. 30.5%; *p* < 0.001). Men required non-invasive mechanical ventilation (NIVM) and invasive mechanical ventilation (IMV) more frequently than women (5.6% vs. 3.6%, *p* < 0.001, and 7.9% vs. 4.8%, *p* < 0.001, respectively). Oxygen via high-flow nasal cannula was also used more frequently in men (9.4% vs. 7.2%, *p* < 0.001) and prone positioning was used in 12.5% of men and 7.2% of women (*p* < 0.001). Full anticoagulant doses of low-molecular-weight heparin (LMWH) were more frequently administered in the male group (11.8% vs. 9.0%; *p* < 0.001). The complete data on the treatments, including the other types of treatments prescribed during hospitalization, can be seen in Table 3.

### 3.4. Outcomes

Regarding complications during hospitalization, men suffered more complications. Acute respiratory distress syndrome (ARDS) was the most common complication, and severe ARDS was more frequently in men (19.9% vs. 14%, *p* < 0.001). Men needed ICU admission more often than women (10% vs. 6.1%, *p* < 0.001), and presented higher in-hospital mortality (23.1% vs. 18.9%, *p* < 0.001). Table 4 details the data on complications during admission, ICU admission, and in-hospital mortality.

**Figure 2:**
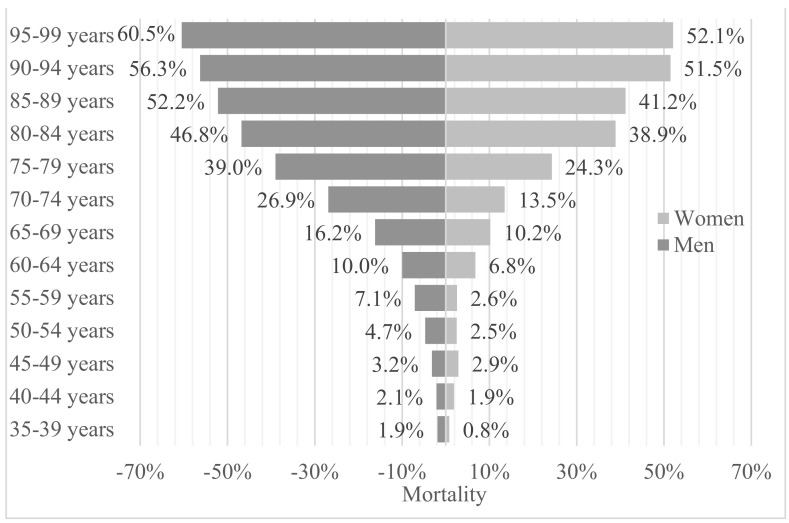
Mortality between the sexes according to age range.

**Figure 3:**
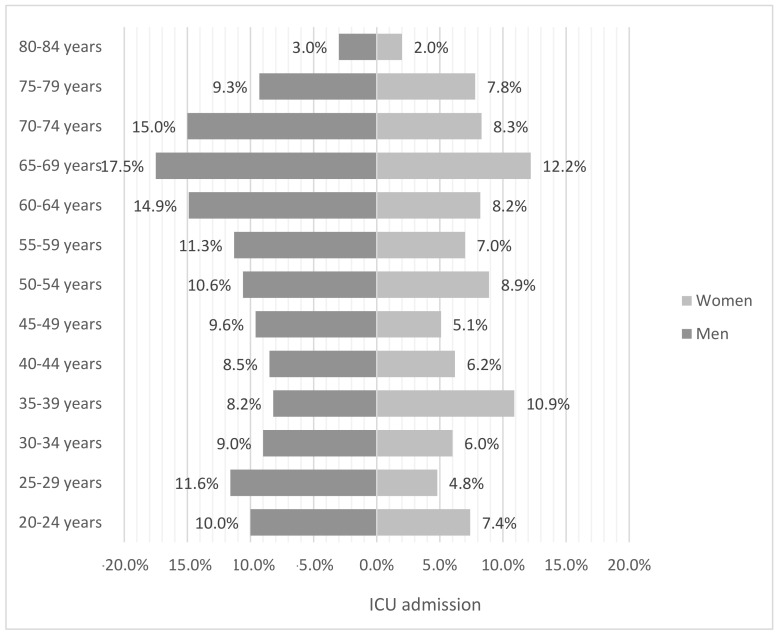
Intensive Care Unit (ICU) admission by sex and age range.

We analyzed the age and sex distribution of the reported COVID-19 deaths in our study, as well as ICU admission by age subgroups. A complete picture of the results can be seen in Figure 2 and Figure 3. In both data sets, men have a higher mortality and income rate in the ICU. Most of the deceased were over 85 years of age, in both groups, men and women (52.2% vs. 41.2%, *p* < 0.001). It is important that when we compare the graphs, the percentages should be related to the size of the population in each subgroup, since it is notable that, as age increases, the gender gap is more noticeable despite the fact that women are in the majority in the upper age groups, and—in our study—also present greater dependence than men. Regarding mortality, the differences by gender are statistically significant in the age groups from 55 years to 89. If we take into account the ICU admissions, the differences are significant from 45 to 74 years, except in the age group of 50 to 54 years. It is important to note that the age group with the greatest difference with a great statistical significance is that of 70 to 74 years (15% vs. 8.3%, *p* < 0.001).

### 3.5. Risk Factors for Mortality

A multivariate analysis was conducted in order to analyze the associations between some of the aforementioned variables and mortality. The variables of age, moderate and severe dependence, a Charlson comorbidity index above 3 points, confusion, tachypnea, radiological worsening at seven days, and severe acute respiratory distress syndrome were independently associated with higher mortality in the multivariate analysis. Therefore, our analysis shows that female sex was significantly and independently associated with a lower risk of mortality in the patients admitted for SARS-COV-2 infection (Hazard Ratio (HR) 0.771; confidence interval (CI) 95% 0.642-0925, *p* = 0.005). In this analysis, fever, dyspnea, and bilateral radiological involvement were not shown to be independent factors of mortality. Table 5 details the multivariate analysis of the selected variables in relation to mortality.

## 4. Discussion

Since the onset of the COVID-19 pandemic, and since the first studies on the disease were conducted, a greater vulnerability to the disease and its complications, as well as a higher mortality rate, have been observed in men compared to women. Along these lines, this work has found that men with COVID-19 had complications more often, had a higher rate of ICU admission, and had a higher mortality rate compared to women.

Our data coincide with those reported in other studies. In Italy, a study comparing different variables that analyzed the effect of COVID-19 on daily deaths per 100,000 people of all genders and ages showed 30% higher mortality in men compared to women, with this difference becoming notably more striking in those aged 60 years and older. Worldwide, it is estimated that twice as many men die due to COVID-19 than women, according to data from the European Center for Disease Prevention and Control [9,10].

In the UK, researchers at the Intensive Care National Audit and Research Centre analyzed a sample of 7542 critically ill patients with confirmed COVID-19 and found that 5389 of these patients were men and 2140 were women. They also found that men were more likely to die in the ICU, with 51% of the men who were admitted dying compared to 43% of the women [11].

One of the first studies on the differences between men and women with COVID-19 was a retrospective analysis of 168 patients, which evaluated the differences between the clinical characteristics of critically ill patients according to sex. Again, its age- and comorbidity-adjusted results also found higher mortality and a worse prognosis among men [12].

In a recent meta-analysis on COVID-19, the authors confirmed the higher mortality of men over women. They placed particular importance on external factors that could contribute to this increase in deaths, especially in terms of habits and lifestyles. For example, one of the works included was a Chinese study in which the prevalence of smoking among men was ten times higher than that in women, which could have a negative impact on their health status. This could therefore entail a prior deteriorated health status that could condition greater severity of infection in men [13,14].

In our work, it is striking that even though the women in the study were older than the men, and had a higher percentage of severe dependence, coupled with the fact that both groups had similar percentages of comorbidities, the men had greater mortality and a higher number of ICU admissions. However, it is true that the men arrived at our hospital in worse condition and with more serious symptoms than the women, and a greater percentage of the men had established bilateral pneumonia upon admission.

Another important finding of our work concerns the differences in the treatments received according to sex. A greater use of systemic treatments and respiratory therapies was observed in the male group compared to the female group. In regard to this finding, we hypothesize that the men in our study were younger than the women, and thus had a higher life expectancy; therefore, they may have been administered more treatments.

Alternatively, another explanation for why men had worse clinical progress than women may be due to reasons that are not yet clearly defined.

It is important to note, in our study, that the differences in mortality in terms of gender are greater and statistically significant according to the older age of the patients. Thus, we find similar data in the literature, as in a study with a sample of patients in New York, in which increasing age and male sex are independent factors associated with worse in-hospital evolution [15].

In another study carried out with a sample of Italian patients, the authors concluded that men from the age of 50 and women from the age of 80 had a worse prognosis in terms of radiological evolution (although the sample of men was almost three times larger than that of women), and if we look at the data of our study, in men, the percentage of higher mortality begins to be notably different from the other age ranges after 55 years, and in women after 75 years, which could overlap with the conclusions of the Italian study [16].

A meta-analysis that included 60 studies and more than 50,000 patients found that age played an important role in the analysis by subgroups, as although advanced age supposes an independent risk of mortality or worse prognosis, if other factors were taken into account, such as smoking or dyspnea on admission, mortality was higher in younger patient groups, and they concluded that these two factors are associated with a worse prognosis in populations with less exposure to the main risk factors for mortality from COVID-19, that is, elderly patients, a high proportion of men, and a high prevalence of comorbidities [17].

In another study with Canadian patients, they compared differences between women and men in different age groups in terms of hospital admission, ICU admission, time to recovery, and mortality, and found that there were large differences in women under 30 years of age and men of the same age in terms of hospitalization and admission to the ICU, and observed that, from the age of 60, there was a significant decrease in mortality in women compared to men of the same age group. They concluded that estrogens in women after puberty and before menopause could play a fundamental role, and that there may be a possible mechanism between estrogen levels and mortality/time to recovery that is not yet well defined, although in women aged over 60 years there would have to be other additional factors [18].

In China, in a study of 80,000 patients, patients older than 60 years had a 9.9 times greater risk of dying than patients younger than 30 years. Regarding the differences by gender, women presented less severity of the disease in all age ranges except those between 20 and 29 years. Regarding mortality, in all age groups, it was lower in women, with this difference being statistically significant from 30 years [19].

In another meta-analysis with 59 studies and more than 36,000 patients included, men demonstrated more severe forms of SARS-CoV-2 disease than women in all age groups. In addition, men had a higher ICU admission and mortality rate than women. In this meta-analysis, patients in both groups (men and women) older than 70 years had a worse prognosis, higher mortality, and a higher rate of admission to the ICU than those younger than 70 years [20].

Another interesting study on laboratory hematological parameters in COVID-19 infection suggests that it is important to carry out studies that analyze the differences in these parameters between men and women, as these results could be valuable to determine why the disease manifests itself in men more seriously than in women. In our study, men had higher lymphopenia and higher lactate dehydrogenase (LDH) and D-dimer levels, with these results being statistically significant [21].

This leads us to wonder whether men have worse progress solely due to their sex, or whether there are other intervening factors. Some possible factors can be observed in the multivariate analysis conducted in this study, such as older age, radiological worsening at seven days, and confusion at the onset of the disease. Indeed, our data show that men develop more symptoms—such as a cough, fever, and tachypnea—at the onset of the disease than women.

Delving deeper into the influence that comorbidities and lifestyle can have on COVID-19 infection according to gender, one study in the literature examined the differences between females and males among COVID-19 patients in China. In this work, the men were more likely than the women to have two or more major comorbidities. Of those with COVID-19 and chronic obstructive pulmonary disease, 83.3% were men. Males represented 58.9% of those with diabetes, and 62.1% of those with some type of cardiovascular disease. Several studies have shown that, in places where mortality is higher in men than in women, men largely show behaviors that can worsen health, such as smoking, poor diet, or a sedentary lifestyle, along with their related comorbidities. Indeed, in our study, more men were smokers than women. An analysis of other lifestyle factors that were not assessed in our registry would be an interesting future line of research [22,23,24].

Finally, there are studies that attempt to explain this bias between men and women according to physiopathogenesis and innate characteristics of biological sex. In the literature consulted, we found several articles that defend the notion that women have a better immune response to external pathogens, and many of the genes that regulate this immune response are found on the X chromosome. Hormones could also play a fundamental role; estrogens could be beneficial in improving the immune response, as they enhance the proliferation of T lymphocytes and attenuate the cytokine storm, while testosterone in men could have a certain negative effect [25,26].

Another study by Haitao et al. on the mechanisms by which biological sex can influence the prognosis of COVID-19 disease concluded that the greater severity and mortality in men compared to women is most likely due to a combination of factors: on the one hand, differences regarding behavior and lifestyles and, on the other hand, comorbidities that affect men to a greater extent. They conclude that biological sex differences could affect the risk of contracting the disease, its physiopathogenic mechanisms, and its severity due to, among other causes, the fact that men are more likely to develop the dreaded cytokine storm associated with a higher lethality of the virus [27].

Another possible explanation is that sex differences in COVID-19 could be related to different angiotensin converting enzyme 2 (ACE2) receptor expression, as several studies have shown a greater presence of ACE2 receptor receptors in the Leydig cells and kidneys of men due to mechanisms possibly explained by hormonal differences. This is why diffuse alveolar damage is the main cause of morbidity and mortality in SARS-CoV-2 infection. Older age is also related to a higher expression of ACE2 receptors, although a greater number of specific studies are necessary in order to be able to develop a definitive theory. A study that began before the COVID-19 pandemic which aimed to investigate patients with heart failure and ACE2 found that men, older patients, and those with more comorbidities had higher concentrations of ACE2. These results were validated with another control group of more than 2000 patients. It was suggested that, perhaps, this was the reason why men are more susceptible to contracting the infection and presenting with worsening symptoms than women [26,28].

The data in this study are from the ‘first wave’ of contagions in Spain. Today, the SARS-CoV-2 epidemic continues to progress exponentially, and it is necessary to learn from past mistakes in order to improve the treatment of this disease. It is clear that some gender-related aspects—such as comorbidities or lifestyle choices like active smoking, which are more common among males—refuted in our study may play a key role in the higher percentage of ICU admission and higher mortality of men compared to women, although other circumstances related to biological sex that are not analyzed in our registry should be taken into account as possible endpoints in future studies.

The strength of this study is that it is a multicenter study which included a large number of patients. On the other hand, there are limitations, as statistically more complete and advanced studies with larger sample sizes are required due to the overwhelming incidence of the disease. Furthermore, the retrospective nature of the study, and the fact that the vast majority of the patients in this study were Caucasian, that all required hospital admission, and the unknown real spread of COVID-19 among outpatients, mean that our results cannot be easily extrapolated to the general population.

## 5. Conclusions

Male sex seems to be related to worse progress in the SARS-CoV-2 disease. Men present with more symptoms at the disease’s onset, have more complications during hospitalization, require a greater number of treatments, are more frequently admitted to the ICU, and have a higher mortality rate. Male sex and older age seem to be independent prognostic factors for mortality, but—in order to fully understand its impact on the prognosis of the disease—we must always consider other factors associated with sex.

## Figures and Tables

**Table 1:** Baseline characteristics and comorbidities of the study patients.

	Male *n* = 6853 (56.8%)	Female *n* = 5210 (43.2%)	Total *n* = 12063	*p*-Value
Age, mean (SD)	65.7 (15.8)	67.9 (16.9)	66.7 (16.3)	<**0.001**
Smoker, *n* (%)	445 (6.9)	167 (3.3)	612 (5.3)	<**0.001**
Obesity, *n* (%)	1265 (20.5)	1052 (22.3)	2317 (21.2)	**0.022**
Arterial hypertension, *n* (%)	3422 (50.0)	2636 (50.7)	6058 (50.3)	0.453
Dyslipidemia, *n* (%)	2699 (39.5)	2052 (39.5)	4751 (39.5)	0.997
Atrial fibrillation, *n* (%)	786 (11.5)	554 (10.7)	1340 (11.1)	0.145
Severe dependency, *n* (%)	346 (5.1)	516 (10.1)	862 (7.3)	<**0.001**
Charlson Comorbidity Index, mean (SD)	3.57 (2.76)	3.59 (2.60)	3.58 (2.69)	0.729
Anxiety, *n* (%)	323 (4.7)	636 (12.3)	959 (8.0)	<**0.001**
Depression, *n* (%)	418 (6.1)	844 (16.3)	1262 (10.5)	<**0.001**
Neurodegenerative disease, *n* (%)	524 (7.7)	606 (11.7)	1130 (9.4)	<**0.001**
Rare disease, *n* (%)	111 (1.6)	90 (1.7)	201 (1.7)	<**0.001**
Dialysis, *n* (%)	79 (1.2)	43 (0.8)	122 (1.0)	0.109

SD: standard deviation.

**Table 2:** Clinical presentation.

	Male *n* = 6853 (56.8)	Female *n* = 5210 (43.2)	Total *n* = 12,063	*p*-Value
**Symptoms**				
Dry cough, *n* (%)	3996 (58.5)	3011 (58.1)	7007 (58.3)	<**0.001**
Productive cough, *n* (%)	1152 (16.9)	723 (13.9)	1875 (15.6)	
Fever > 38°, *n* (%)	4650 (68.1)	2976 (57.4)	7626 (63.5)	<**0.001**
Ageusia, *n* (%)	414 (6.2)	381 (7.6)	795 (6.8)	**0.005**
Anorexia, *n* (%)	1267 (18.9)	1063 (20.9)	2330 (19.8)	**0.008**
Headache, *n* (%)	729 (10.8)	635 (12.4)	1364 (11.5)	**0.007**
Diarrhea, *n* (%)	1473 (21.7)	1285 (24.9)	2758 (23.1)	<**0.001**
General malaise, *n* (%)	663 (9.9)	805 (15.9)	1468 (12.5)	<**0.001**
Vomiting, *n* (%)	377 (5.6)	506 (9.9)	883 (7.4)	<**0.001**
Abdominal pain, *n* (%)	376 (5.6)	402 (7.8)	778 (6.5)	<**0.001**
Arthralgia, *n* (%)	2043 (30.2)	1610 (31.5)	3653 (30.8)	0.158
Anosmia, *n* (%)	385 (5.8)	331 (6.6)	716 (6.1)	0.089
Asthenia, *n* (%)	2878 (42.7)	2266 (44.4)	5144 (43.4)	0.072
Odynophagia, *n* (%)	631 (9.4)	531 (10.4)	1162 (9.8)	0.062
Dyspnea, *n* (%)	3971 (58.2)	2960 (57.2)	6931 (57.8)	0.265
**Physical Examination**				
Confusion, *n* (%)	713 (10.6)	702 (13.7)	1415 (11.9)	<**0.001**
Tachypnea, *n* (%)	2173 (32.6)	1505 (29.8)	3678 (31.4)	**0.002**
Hypotension (SBP < 100 mmHg), *n* (%)	358 (5.5)	331 (6.7)	689 (6.0)	**0.006**
Tachycardia (>100 bpm), *n* (%)	1502 (22.8)	1075 (21.5)	2577 (22.2)	0.103
Oxygen saturation < 92%, *n* (%)	2363 (35.6)	1569 (31.1)	3932 (33.6)	<**0.001**
Wheezing, *n* (%)	355 (5.3)	359 (7.1)	714 (6.1)	<**0.001**
Rales, *n* (%)	757 (11.4)	517 (10.2)	1274 (10.9)	**0.045**
Crackles, *n* (%)	3504 (52.8)	2648 (52.4)	6157 (52.7)	0.666
**Radiological Data**				
Unilateral condensation, *n* (%) Bilateral condensation, *n* (%)	1224 (18.0) 2158 (31.8)	885 (17.3) 1526 (29.9)	2109 (17.7) 3684 (31.0)	0.071 **0.007**
Unilateral interstitial infiltrates, *n* (%) Bilateral interstitial infiltrates, *n* (%)	700 (10.3) 3647 (53.7)	547 (10.7) 2481 (48.6)	1247 (10.5) 6128 (51.5)	0.194 <**0.001**
Unilateral pleural effusion, *n* (%) Bilateral pleural effusion, *n* (%)	192 (2.8) 89 (1.3)	152 (3.0) 96 (1.9)	344 (2.9) 185 (1.6)	0.587 **0.012**
Radiological worsening *, *n* (%)	2255 (43.6)	1343 (35.9)	3598 (40.4)	<**0.001**
**Laboratory Data**				
Lymphocytes < 1500/mm3, *n* (%)	5771 (84.8)	4101 (79.9)	9872 (82.7)	<**0.001**
LDH > 300 IU/L, *n* (%)	3377 (56.9)	2255 (51.1)	5632 (54.4)	<**0.001**
D-Dimer > 500 ng/mL, *n* (%)	3148 (59.7)	2417 (61.5)	5565 (60.4)	0.084

* Radiological worsening at follow-up at 7 days, discharge, or death. LDH: lactate dehydrogenase; SBP: systolic blood pressure. bpm: beats per min. mmHg: millimeters of mercury.

**Table 3:** Treatment during admission.

	Male *n* = 6853 (56.8)	Female *n* = 5210 (43.2)	Total *n* = 12,063	*p*-Value
**Most Used Treatments**				
Hydroxychloroquine, *n* (%)	5931 (86.9)	4353 (84.1)	10,284 (85.7)	<**0.001**
Lopinavir/ritonavir, *n* (%)	4438 (65.1)	2954 (57.1)	7392 (61.7)	<**0.001**
Remdesivir, *n* (%)	42 (0.6)	16 (0.3)	58 (0.5)	**0.016**
Interferon beta-1B, *n* (%)	943 (13.9)	481 (9.4)	1424 (11.9)	<**0.001**
Tocilizumab, *n* (%)	738 (10.9)	324 (6.3)	1062 (8.9)	<**0.001**
Systemic steroids, *n* (%)	2632 (38.7)	1571 (30.5)	4203 (35.2)	<**0.001**
Chloroquine, *n* (%)	313 (4.6)	215 (4.2)	528 (4.4)	0.243
Colchicine, *n* (%)	60 (0.9)	45 (0.9)	105 (0.9)	0.959
Immunoglobulins, *n* (%)	35 (0.5)	15 (0.3)	50 (0.4)	0.061
Anakinra, *n* (%)	45 (0.7)	15 (0.3)	60 (0.5)	**0.005**
Baricitinib, *n* (%)	29 (0.6)	11 (0.1)	40 (0.3)	**0.043**
Oseltamivir, *n* (%)	66 (1.0)	36 (0.7)	102 (0.9)	0.109
Inhaled beclomethasone, *n* (%)	390 (5.8)	284 (5.6)	674 (5.7)	0.593
**Antibiotics**				
Beta-lactams, *n* (%)	5188 (76.2)	3642 (70.4)	8830 (73.7)	<**0.001**
Macrolides, *n* (%)	4223 (62.1)	3013 (58.4)	7236 (60.0)	<**0.001**
Quinolones, *n* (%)	894 (13.3)	645 (12.6)	1539 (13.0)	0.268
**Respiratory Therapies**				
NIMV, *n* (%)	384 (5.6)	187 (3.6)	571 (4.8)	<**0.001**
IMV, *n* (%)	541 (7.9)	249 (4.8)	790 (6.6)	<**0.001**
High flow nasal cannula, *n* (%)	640 (9.4)	368 (7.2)	1008 (8.5)	<**0.001**
Prone positioning, *n* (%)	851 (12.5)	370 (7.2)	1221 (10.2)	<**0.001**
**Other Treatments During Admission**				
ACEI, *n* (%)	672 (9.9)	421 (8.2)	1093 (9.2)	**0.001**
ARB, *n* (%)	703 (10.4)	535 (10.4)	1238 (10.4)	0.965
ASA, *n* (%)	962 (14.3)	581 (11.4)	1543 (13.1)	<**0.001**
Statins, *n* (%)	967 (14.4)	642 (12.6)	1609 (13.6)	**0.005**
Vitamin K antagonists, *n* (%) DOAC, *n* (%)	109 (1.6) 127 (1.9)	91 (1.8) 80 (1.6)	200 (1.7) 207 (1.7)	0.516 0.190
LMWH: Prophylactic doses, *n* (%) LMWH: Full anticoagulant doses, *n* (%) LMWH: Intermediate doses, *n* (%)	4354 (64.2) 797 (11.8) 500 (7.4)	3435 (66.7) 464 (9.0) 294 (5.7)	7789 (65.3) 1261 (10.6) 794 (6.7)	0.139 <**0.001** <**0.001**
Ibuprofen, *n* (%) Other NSAIDs, *n* (%)	65 (1.0) 285 (4.2)	46 (0.9) 215 (4.2)	111 (0.9) 500 (4.2)	0.771 0.934

NIMV: non-invasive mechanical ventilation; IMV: invasive mechanical ventilation; ACEI: angiotensin converting enzyme inhibitors; ARB: angiotensin ii receptor blockers; ASA: acetylsalicylic acid; DOAC: direct oral anticoagulant; LMWH: low molecular weight heparin; NSAIDs: nonsteroidal anti-inflammatory drugs.

**Table 4:** Complications, ICU admission rate, and in-hospital mortality.

	Male *n* = 6853 (56.8%)	Female *n* = 5210 (43.2%)	Total *n* = 12,063	*p*-Value
**Complications**				
ARDS mild, *n* (%) ARDS moderate, *n* (%) ARDS severe, *n* (%)	602 (8.9) 570 (8.4) 1352 (19.9)	380 (7.4) 311 (6.0) 722 (14.0)	982 (8.2) 881 (7.4) 2074 (17.4)	<**0.001** <**0.001** <**0.001**
Acute kidney injury, *n* (%)	1082 (15.9)	609 (11.8)	1691 (14.1)	<**0.001**
Bacterial pneumonia, *n* (%)	806 (11.9)	480 (9.3)	1286 (10.8)	<**0.001**
Multi-organic failure, *n* (%)	505 (7.4)	252 (4.9)	757 (6.3)	<**0.001**
Sepsis, *n* (%)	481 (7.1)	267 (5.2)	748 (6.3)	<**0.001**
Shock, *n* (%)	366 (5.4)	173 (3.4)	539 (4.5)	<**0.001**
DIC, *n* (%)	89 (1.3)	41 (0.8)	130 (1.1)	**0.007**
Acute myocardial infarction, *n* (%)	67 (1.0)	28 (0.5)	95 (0.8)	**0.007**
Peripheral arterial disease, *n* (%)	43 (0.6)	13 (0.3)	56 (0.5)	**0.003**
Heart failure, *n* (%)	398 (5.9)	333 (6.5)	731 (6.1)	0.176
Atrial arrhythmia, *n* (%) Ventricular arrhythmia, *n* (%)	251 (3.7) 27 (0.4)	179 (3.5) 9 (0.2)	429 (3.6) 36 (0.3)	0.462 **0.031**
Myocarditis, *n* (%)	74 (1.1)	43 (0.8)	117 (1.0)	0.159
Seizures, *n* (%)	38 (0.3)	34 (0.3)	72 (0.6)	0.484
Ischemic stroke, *n* (%) Hemorrhagic stroke, *n* (%)	36 (0.5) 7 (0.1)	30 (0.6) 1 (0)	66 (0.6) 8 (0.1)	0.712 0.149
VTE: DVT, *n* (%) VTE: PE, *n* (%) VTE: DVT + PE, *n* (%)	29 (0.4) 103 (1.5) 9 (0.1)	23 (0.4) 66 (1.3) 7 (0.1)	52 (0.4) 169 (1.4) 16 (0.1)	0.892 0.281 0.964
**ICU Admission Rate and in-Hospital Mortality**				
ICU admission, *n* (%)	684 (10.0)	318 (6.1)	1002 (8.3)	<**0.001**
In-hospital mortality, *n* (%)	1547 (23.1)	962 (18.9)	2509 (21.3)	<**0.001**

ARDS: acute respiratory distress syndrome; DIC: disseminated intravascular coagulation; VTE: venous thromboembolism; DVT: deep vein thrombosis; PE: pulmonary embolism.

**Table 5:** Independent variables associated with in-hospital death.

Mortality	Univariate Analysis		Multivariate Analysis	
	**HR (CI 95%)**	***p*-Value**	**HR (CI 95%)**	***p*-Value**
Gender (female)	0.777 (0.710–0.851)	<0.001	0.771 (0.642–0.925)	0.005
Age	0.920 (0.916–0.924)	<0.001	0.945 (0.936–0.954)	<0.001
Moderate dependency Severe dependency	5.749 (4.970–6.650) 1.203 (1.005–1.439)	<0.001 0.044	2.808 (2.049–3.847) 1.405 (1.004–1.967)	<0.001 0.047
Charlson Comorbidity Index < 3 points	9.759 (8.668–10.988)	<0.001	3.431 (2.673–4.403)	<0.001
Fever > 38 °C	0.812 (0.727–0.907)	<0.001	0.907 (0.724–1.135)	0.393
Dyspnea	2.097 (1.095–2.308)	<0.001	1.173 (0.968–1.422)	0.104
Confusion	6.321 (5.619–7.111)	<0.001	2.264 (1.781–2.877)	<0.001
Tachypnea > 20 bpm	4.049 (3.687–4.446)	<0.001	1.321 (1.096–1.592)	0.003
Severe ARDS	4.757 (4.015–5.638)	<0.001	7.942 (6.084–10.366)	<0.001
Bilateral pneumonia	1.386 (1.216–1.579)	<0.001	1.168 (0.898–1.519)	0.247
Radiological worsening at 7 days of hospitalization	5.003 (4.424–5.658)	<0.001	2.352 (1.956–2.828)	<0.001

ARDS: acute respiratory distress syndrome.

## Data Availability

Data is contained within the article.

## Appendix A

**Table A1:** List of the SEMI-COVID-19 Network members.

Anxela Crestelo Vieitez	anxela90@gmail.com
María del Mar García Andreu	mariadelmargarciaandreu@gmail.com
Claudia Josa Laorden	claudiajosa@gmail.com
Manuel Rubio-Rivas	mrubio@bellvitgehospital.cat
Marcos Sánchez	marcossanferab@gmail.com
Neera Toledo Samaniego	neera.toledo@salud.madrid.org
Francisco Arnalich Fernández	farnalich@salud.madrid.org
Rosario Iguaran Bermudez	draiguaran@gmail.com
Eva Mª Fonseca Aizpuru	evamfonseca@yahoo.es
Juan Antonio Vargas Núñez	juanantonio.vargas@uam.es
Paula Maria Pesqueira Fontan	paulapesqueira@hotmail.com
Jorge Serrano Ballesteros	jserrano645@gmail.com
Santiago Jesús Freire Castro	santiago.freire.castro@sergas.es
Melani Pestaña Fernández	Melani.Pestana@sanitatintegral.org
Alba Viana García	alvigar83@gmail.com
Victoria Nuñez Rodriguez	mijas29@hotmail.com
Vicente Giner-Galvañ	ginervicgal@gmail.com
Francisco Javier Carrasco Sánchez	fjcarrascos@icloud.com
Almudena Hernández Milián	ahernandez4@hsll.es
Marta Cobos-Siles	mcoboss@saludcastillayleon.es
Jose Javier Napal Lecumberri	josejavier.napal@scsalud.es
Virginia Herrero García	virgyherrero@hotmail.com
Maria de los Reyes Pascual Pérez	cperezb@coma.es
Jesús Millán Núñez-Cortés	jesus.millan@salud.madrid.org
José Manuel Casas Rojo	jm.casas@gmail.com

Coordinator of the SEMI-COVID-19 Registry: José Manuel Casas Rojo.

**SEMI-COVID-19 Scientific Committee Members:** José Manuel Casas Rojo, José Manuel Ramos Rincón, Carlos Lumbreras Bermejo, Jesús Millán Núñez-Cortés, Juan Miguel Antón Santos, Ricardo Gómez Huelgas.

SEMI-COVID-19 Registry Coordinating Center: S & H Medical Science Service.

Members of the SEMI-COVID-19 Group

Hospital Universitari de Bellvitge. L’Hospitalet de Llobregat

Xavier Corbella, Narcís Homs, Abelardo Montero, Jose María Mora-Luján, Manuel Rubio-Rivas.

H. U. 12 de Octubre. Madrid

Paloma Agudo de Blas, Coral Arévalo Cañas, Blanca Ayuso, José Bascuñana Morejón, Samara Campos Escudero, María Carnevali Frías, Santiago Cossio Tejido, Borja de Miguel Campo, Carmen Díaz Pedroche, Raquel Diaz Simon, Ana García Reyne, Lucia Jorge Huerta, Antonio Lalueza Blanco, Jaime Laureiro Gonzalo, Jaime Lora-Tamayo, Carlos Lumbreras Bermejo, Guillermo Maestro de la Calle, Barbara Otero Perpiña, Diana Paredes Ruiz, Marcos Sánchez Fernández, Javier Tejada Montes.

H. U. Gregorio Marañón. Madrid

Laura Abarca Casas, Álvaro Alejandre de Oña, Rubén Alonso Beato, Leyre Alonso Gonzalo, Jaime Alonso Muñoz, Crhistian Mario Amodeo Oblitas, Cristina Ausín García, Marta Bacete Cebrián, Jesús Baltasar Corral, Maria Barrientos Guerrero, Alejandro Bendala Estrada, María Calderón Moreno, Paula Carrascosa Fernández, Raquel Carrillo, Sabela Castañeda Pérez, Eva Cervilla Muñoz, Agustín Diego Chacón Moreno, Maria Carmen Cuenca Carvajal, Sergio de Santos, Andrés Enríquez Gómez, Eduardo Fernández Carracedo, María Mercedes Ferreiro-Mazón Jenaro, Francisco Galeano Valle, Alejandra Garcia, Irene Garcia Fernandez-Bravo, María Eugenia García Leoni, María Gómez Antúnez, Candela González San Narciso, Anthony Alexander Gurjian, Lorena Jiménez Ibáñez, Cristina Lavilla Olleros, Cristina Llamazares Mendo, Sara Luis García, Víctor Mato Jimeno, Clara Millán Nohales, Jesús Millán Núñez-Cortés, Sergio Moragón Ledesma, Antonio Muiño Míguez, Cecilia Muñoz Delgado, Lucía Ordieres Ortega, Susana Pardo Sánchez, Alejandro Parra Virto, María Teresa Pérez Sanz, Blanca Pinilla Llorente, Sandra Piqueras Ruiz, Guillermo Soria Fernández-Llamazares, María Toledano Macías, Neera Toledo Samaniego, Ana Torres do Rego, Maria Victoria Villalba Garcia, Gracia Villarreal, María Zurita Etayo.

H. U. La Paz-Cantoblanco-Carlos III. Madrid

Jorge Álvarez Troncoso, Francisco Arnalich Fernández, Francisco Blanco Quintana, Carmen Busca Arenzana, Sergio Carrasco Molina, Aranzazu Castellano Candalija, Germán Daroca Bengoa, Alejandro de Gea Grela, Alicia de Lorenzo Hernández, Alejandro Díez Vidal, Carmen Fernández Capitán, Maria Francisca García Iglesias, Borja González Muñoz, Carmen Rosario Herrero Gil, Juan María Herrero Martínez, Víctor Hontañón, Maria Jesús Jaras Hernández, Carlos Lahoz, Cristina Marcelo Calvo, Juan Carlos Martín Gutiérrez, Monica Martinez Prieto, Elena Martínez Robles, Araceli Menéndez Saldaña, Alberto Moreno Fernández, Jose Maria Mostaza Prieto, Ana Noblejas Mozo, Carlos Manuel Oñoro López, Esmeralda Palmier Peláez, Marina Palomar Pampyn, Maria Angustias Quesada Simón, Juan Carlos Ramos Ramos, Luis Ramos Ruperto, Aquilino Sánchez Purificación, Teresa Sancho Bueso, Raquel Sorriguieta Torre, Clara Itziar Soto Abanedes, Yeray Untoria Tabares, Marta Varas Mayoral, Julia Vásquez Manau.

H. Clínico San Carlos. Madrid

Inés Armenteros Yeguas, Javier Azaña Gómez, Julia Barrado Cuchillo, Irene Burruezo López, Noemí Cabello Clotet, Alberto E. Calvo Elías, Elpidio Calvo Manuel, Carmen María Cano de Luque, Cynthia Chocron Benbunan, Laura Dans Vilan, Claudia Dorta Hernández, Ester Emilia Dubon Peralta, Vicente Estrada Pérez, Santiago Fernandez-Castelao, Marcos Oliver Fragiel Saavedra, José Luis García Klepzig, Maria del Rosario Iguarán Bermúdez, Esther Jaén Ferrer, Alejandro Maceín Rodríguez, Alejandro Marcelles de Pedro, Rubén Ángel Martín Sánchez, Manuel Méndez Bailón, Sara Miguel Álvarez, Maria José Nuñez Orantos, Carolina Olmos Mata, Eva Orviz García, David Oteo Mata, Cristina Outon González, Juncal Perez-Somarriba, Pablo Pérez Mateos, Maria Esther Ramos Muñoz, Xabier Rivas Regaira, Laura Mª Rodríguez Gallardo, Iñigo Sagastagoitia Fornie, Alejandro Salinas Botrán, Miguel Suárez Robles, Maddalena Elena Urbano, Andrea María Vellisca González, Miguel Villar Martínez.

H. de Cabueñes. Gijón

Ana María Álvarez Suárez, Carlos Delgado Vergés, Rosa Fernandez-Madera Martínez, Eva Mª Fonseca Aizpuru, Alejandro Gómez Carrasco, Cristina Helguera Amezua, Juan Francisco López Caleya, María del Mar Martínez López, Aleida Martínez Zapico, Carmen Olabuenaga Iscar, María Luisa Taboada Martínez, Lara María Tamargo Chamorro.

Hospital Royo Villanova. Zaragoza

Nicolás Alcalá Rivera, Anxela Crestelo Vieitez, Esther del Corral Beamonte, Jesús Díez Manglano, Isabel Fiteni Mera, Maria del Mar Garcia Andreu, Martin Gericó Aseguinolaza, Claudia Josa Laorden, Raul Martínez Murgui, Marta Teresa Matía Sanz.

Hospital Clínico de Santiago. Santiago de Compostela

Maria del Carmen Beceiro Abad, Maria Aurora Freire Romero, Sonia Molinos Castro, Emilio Manuel Paez Guillan, María Pazo Nuñez, Paula Maria Pesqueira Fontan.

H. U. Puerta de Hierro. Majadahonda

María Álvarez Bello, Ane Andrés Eisenhofer, Ana Arias Milla, Isolina Baños Pérez, Laura Benítez Gutiérrez, Javier Bilbao Garay, Silvia Blanco Alonso, Jorge Calderón Parra, Alejandro Callejas Díaz, José María Camino Salvador, Mª Cruz Carreño Hernández, Valentín Cuervas-Mons Martínez, Sara de la Fuente Moral, Miguel del Pino Jimenez, Alberto Díaz de Santiago, Itziar Diego Yagüe, Ignacio Donate Velasco, Ana María Duca, Pedro Durán del Campo, Gabriela Escudero López, Esther Expósito Palomo, Ana Fernández Cruz, Esther Fiz Benito, Andrea Fraile López, Amy Galán Gómez, Sonia García Prieto, Claudia García Rodríguez-Maimón, Miguel Ángel García Viejo, Javier Gómez Irusta, Edith Vanessa Gutiérrez Abreu, Isabel Gutiérrez Martín, Ángela Gutiérrez Rojas, Andrea Gutiérrez Villanueva, Jesús Herráiz Jiménez, Pedro Laguna del Estal, Mª Carmen Máinez Sáiz, Cristina Martín Martín, María Martínez Urbistondo, Fernando Martínez Vera, Susana Mellor Pita, Patricia Mills Sánchez, Esther Montero Hernández, Alberto Mora Vargas, Cristina Moreno López, Alfonso Ángel-Moreno Maroto, Victor Moreno-Torres Concha, Ignacio Morrás De La Torre, Elena Múñez Rubio, Rosa Muñoz de Benito, Ana Muñoz Gómez, Alejandro Muñoz Serrano, Jose María Palau Fayós, Lina Marcela Parra Ramírez, Ilduara Pintos Pascual, Arturo José Ramos Martín-Vegue, Antonio Ramos Martínez, Isabel Redondo Cánovas del Castillo, Alberto Roldán Montaud, Lucía Romero Imaz, Yolanda Romero Pizarro, Enrique Sánchez Chica, David Sánchez Órtiz, Mónica Sánchez Santiuste, Patricia Serrano de la Fuente, Pablo Tutor de Ureta, Ángela Valencia Alijo, Mercedes Valentín-Pastrana Aguilar, Juan Antonio Vargas Núñez, Jose Manuel Vázquez Comendador, Gema Vázquez Contreras, Carmen Vizoso Gálvez.

H. U. La Princesa. Madrid

María Aguilera García, Ester Alonso Monge, Jesús Álvarez Rodríguez, Claudia Alvarez Varela, Miquel Berniz Gòdia, Marta Briega Molina, Marta Bustamante Vega, Jose Curbelo, Alicia de las Heras Moreno, Ignacio Descalzo Godoy, Alexia Constanza Espiño Alvarez, Ignacio Fernández Martín-Caro, Alejandra Franquet López-Mosteiro, Gonzalo Galvez Marquez, María José García Blanco, Yaiza García del Álamo Hernández, Clara García-Rayo Encina, Noemí Gilabert González, Carolina Guillamo Rodríguez, Nicolás Labrador San Martín, Manuel Molina Báez, Carmen Muñoz Delgado, Pedro Parra Caballero, Javier Pérez Serrano, Laura Rabes Rodríguez, Pablo Rodríguez Cortés, Carlos Rodriguez Franco, Emilia Roy-Vallejo, Monica Rueda Vega, Aresio Sancha Lloret, Beatriz Sánchez Moreno, Marta Sanz Alba, Jorge Serrano Ballesteros, Alba Somovilla, Carmen Suarez Fernández, Macarena Vargas Tirado, Almudena Villa Marti.

H. U. de A Coruña. A Coruña

Alicia Alonso Álvarez, Olaya Alonso Juarros, Ariadna Arévalo López, Carmen Casariego Castiñeira, Ana Cerezales Calviño, Marta Contreras Sánchez, Ramón Fernández Varela, Santiago J. Freire Castro, Ana Padín Trigo, Rafael Prieto Jarel, Fátima Raad Varea, Ignacio Ramil Freán, Laura Ramos Alonso, Francisco Javier Sanmartín Pensado, David Vieito Porto.

H. Moisès Broggi. Sant Joan Despí

Judit Aranda Lobo, Lucía Feria Casanovas, Jose Loureiro Amigo, Miguel Martín Fernández, Isabel Oriol Bermúdez, Melani Pestaña Fernández, Nicolas Rhyman, Nuria Vázquez Piqueras.

Hospital Universitario Dr. Peset. Valencia

Juan Alberto Aguilera Ayllón, Arturo Artero, María del Mar Carmona Martín, María José Fabiá Valls, Maria de Mar Fernández Garcés, Ana Belén Gómez Belda, Ian López Cruz, Manuel Madrazo López, Elisabeth Mateo Sanchis, Jaume Micó Gandia, Laura Piles Roger, Adela Maria Pina Belmonte, Alba Viana García.

Hospital Costa del Sol. Marbella

Victoria Augustín Bandera, Javier García Alegría, Nicolás Jiménez-García, Jairo Luque del Pino, María Dolores Martín Escalante, Francisco Navarro Romero, Victoria Nuñez Rodriguez.

H. Nuestra Señora del Prado. Talavera de la Reina

Sonia Casallo Blanco, Jeffrey Oskar Magallanes Gamboa, Cristina Salazar Mosteiro, Andrea Silva Asiain.

H. U. Ramón y Cajal. Madrid

Luis Fernando Abrego Vaca, Ana Andréu Arnanz, Octavio Arce García, Marta Bajo González, Pablo Borque Sanz, Alberto Cozar Llisto, Sonia de Pedro Baena, Beatriz Del Hoyo Cuenda, Martin Fabregate-Fuente, María Alejandra Gamboa Osorio, Isabel García Sánchez, Andrés González García, Oscar Alberto López Cisneros, Luis Manzano, Miguel Martínez-Lacalzada, Borja Merino Ortiz, Jimena Rey-García, Elisa Riera González, Cristina Sánchez Díaz, Grisell Starita Fajardo, Cecilia Suárez Carantoña, Adrian Viteri-Noël, Svetlana Zhilina Zhilina.

H. U. San Juan de Alicante. San Juan de Alicante

Marisa Asensio Tomás, David Balaz, David Bonet Tur, Ruth Cañizares Navarro, Paloma Chazarra Pérez, Jesús Corbacho Redondo, Eliana Damonte White, Leticia Espinosa Del Barrio, Pedro Jesús Esteve Atiénzar, Carles García Cervera, David Francisco García Núñez, Vicente Giner Galvañ, Angie Gómez Uranga, Javier Guzmán Martínez, Isidro Hernández Isasi, Lourdes Lajara Villar, Verónica Martínez Sempere, Juan Manuel Núñez Cruz, Sergio Palacios Fernández, Juan Jorge Peris García, Andrea Riaño Pérez, José Miguel Seguí Ripoll, Azucena Sempere Mira, Philip Wikman-Jorgensen.

H. Virgen de la Salud. Toledo

Ana Maria Alguacil Muñoz, Marta Blanco Fernández, Veronica Cano, Ricardo Crespo Moreno, Fernando Cuadra Garcia-Tenorio, Blanca Díaz-Tendero Nájera, Raquel Estévez González, María Paz García Butenegro, Alberto Gato Díez, Verónica Gómez Caverzaschi, Piedad María Gómez Pedraza, Julio González Moraleja, Raúl Hidalgo Carvajal, Patricia Jiménez Aranda, Raquel Labra González, Áxel Legua Caparachini, Pilar Lopez Castañeyra, Agustín Lozano Ancin, Jose Domingo Martin Garcia, Cristina Morata Romero, María Jesús Moya Saiz, Helena Moza Moríñigo, Gemma Muñiz Nicolás, Enriqueta Muñoz Platon, Filomena Oliveri, Elena Ortiz Ortiz, Raúl Perea Rafael, Pilar Redondo Galán, María Antonia Sepulveda Berrocal, Vicente Serrano Romero de Ávila, Pilar Toledano Sierra, Yamilex Urbano Aranda, Jesús Vázquez Clemente, Carmen Yera Bergua.

Hospital Regional Universitario de Málaga. Málaga

Ma Mar Ayala-Gutiérrez, Rosa Bernal López, José Bueno Fonseca, Verónica Andrea Buonaiuto, Luis Francisco Caballero Martínez, Lidia Cobos Palacios, Clara Costo Muriel, Francis de Windt, Ana Teresa Fernandez-Truchaud Christophel, Paula García Ocaña, Ricardo Gómez Huelgas, Javier Gorospe García, Sergio Jansen-Chaparro, Maria Dolores López-Carmona, Pablo López Quirantes, Almudena López Sampalo, Elizabeth Lorenzo-Hernández, Juan José Mancebo Sevilla, Jesica Martín Carmona, Luis Miguel Pérez-Belmonte, Iván Pérez de Pedro, Araceli Pineda-Cantero, Carlos Romero Gómez, Michele Ricci, Jaime Sanz Cánovas.

H. Santa Marina. Bilbao

Maria Areses Manrique, Ainara Coduras Erdozain, Ane Labirua-Iturburu Ruiz.

H. Juan Ramón Jiménez. Huelva

Francisco Javier Bejarano Luque, Francisco-Javier Carrasco-Sánchez, Mercedes de-Sousa-Baena, Jaime Díaz Leal, Aurora Espinar Rubio, Maria Franco Huertas, Juan Antonio García Bravo, Andrés Gonzalez Macías, Encarnación Gutiérrez Jiménez, Alicia Hidalgo Jiménez, Constantino Lozano Quintero, Carmen Mancilla Reguera, Francisco Javier Martínez Marcos, Francisco Muñoz Beamud, Maria Pérez-Aguilar, Alícia Pérez Jiménez, Virginia Rodríguez Castaño, Alvaro Sánchez de Alcazar del Río, Leire Toscano Ruiz.

H. del Henares. Coslada

Jesús Ballano Rodríguez-Solís, Luis Cabeza Osorio, María del Pilar Fidalgo Montero, Ma Isabel Fuentes Soriano, Erika Esperanza Lozano Rincon, Ana Martín Hermida, Jesus Martinez Carrilero, Jose Angel Pestaña Santiago, Manuel Sánchez Robledo, Patricia Sanz Rojas, Nahum Jacobo Torres Yebes, Vanessa Vento.

H. U. La Fe. Valencia

Dafne Cabañero, María Calabuig Ballester, Pascual Císcar Fernández, Ricardo Gil Sánchez, Marta Jiménez Escrig, Cristina Marín Amela, Laura Parra Gómez, Carlos Puig Navarro, José Antonio Todolí Parra.

Complejo Hospitalario Universitario Ourense. Ourense

Raquel Fernández González, Amara Gonzalez Noya, Carlos Hernández Ceron, Isabel Izuzquiza Avanzini, Ana Latorre Diez, Pablo López Mato, Ana María Lorenzo Vizcaya, Daniel Peña Benítez, Milagros María Peña Zemsch, Lucía Pérez Expósito, Marta Pose Bar, Lara Rey González, Laura Rodrigo Lara

H. de Mataró. Mataró

Raquel Aranega González, Ramon Boixeda, Javier Fernández Fernández, Carlos Lopera Mármol, Marta Parra Navarro, Ainhoa Rex Guzmán, Aleix Serrallonga Fustier.

H. U. Reina Sofía. Córdoba

Antonio Pablo Arenas de Larriva, Pilar Calero Espinal, Javier Delgado Lista, Francisco Fuentes-Jiménez, María Jesús Gómez Vázquez, Jose Jiménez Torres, Laura Limia Pérez, José López-Miranda, Laura Martín Piedra, Marta Millán Orge, Javier Pascual Vinagre, Pablo Pérez-Martinez, María Elena Revelles Vílchez, Angela Rodrigo Martínez, Juan Luis Romero Cabrera, José David Torres-Peña.

C. H. U. de Badajoz. Badajoz

Rafael Aragon Lara, Inmaculada Cimadevilla Fernandez, Juan Carlos Cira García, Gema Maria García García, Julia Gonzalez Granados, Beatriz Guerrero Sánchez, Francisco Javier Monreal Periáñez, Maria Josefa Pascual Perez.

H. U. Son Llàtzer. Palma de Mallorca

Andrés de la Peña Fernández, Almudena Hernández Milián

H. U. Río Hortega. Valladolid

Irene Arroyo Jiménez, Marina Cazorla González, Marta Cobos-Siles, Luis Corral-Gudino, Pablo Cubero Morais, María González Fernández, José Pablo Miramontes González, Marina Prieto Dehesa, Pablo Sanz Espinosa.

Hospital Alto Guadalquivir. Andújar

Begoña Cortés Rodríguez.

Hospital Infanta Margarita. Cabra

María Esther Guisado Espartero, Lorena Montero Rivas, Maria de la Sierra Navas Alcántara, Raimundo Tirado-Miranda.

C. H. U. de Ferrol. Ferrol

Hortensia Alvarez Diaz, Tamara Dalama Lopez, Estefania Martul Pego, Carmen Mella Pérez, Ana Pazos Ferro, Sabela Sánchez Trigo, Dolores Suarez Sambade, Maria Trigas Ferrin, Maria del Carmen Vázquez Friol, Laura Vilariño Maneiro.

H. de Pozoblanco. Pozoblanco

José Nicolás Alcalá Pedrajas, Antonia Márquez García, Inés Vargas.

Hospital Marina Baixa. Villajoyosa

Javier Ena, Jose Enrique Gómez Segado.

H. U. Virgen de las Nieves. Granada

Pablo Conde Baena, Joaquin Escobar Sevilla, Laura Gallo Padilla, Patricia Gómez Ronquillo, Pablo González Bustos, María Navío Botías, Jessica Ramírez Taboada, Mar Rivero Rodríguez.

Hospital del Tajo. Aranjuez

Ruth Gonzalez Ferrer, Virginia Gracia Lorenzo, Raquel Monsalvo Arroyo.

Hospital Doctor José Molina Orosa. Arrecife (Lanzarote)

Virginia Herrero García, Berta Román Bernal.

H. U. Marqués de Valdecilla. Santander

Marta Fernández-Ayala Novo, José Javier Napal Lecumberri, Nuria Puente Ruiz, Jose Riancho, Isabel Sampedro Garcia.

H. U. Severo Ochoa. Leganés

Yolanda Casillas Viera, Lucía Cayuela Rodríguez, Carmen de Juan Alvarez, Gema Flox Benitez, Laura García Escudero, Juan Martin Torres, Patricia Moreira Escriche, Susana Plaza Canteli, M Carmen Romero Pérez.

H. General Defensa. Zaragoza

Anyuli Gracia Gutiérrez, Leticia Esther Royo Trallero

Hospital Platón. Barcelona

Ana Suarez Lombraña

Hospital Valle del Nalón. Riaño (Langreo)

Sara Fuente Cosío, César Manuel Gallo Álvaro, Julia Lobo García, Antía Pérez Piñeiro.

H. U. del Vinalopó. Elche

Francisco Amorós Martínez, Erika Ascuña Vásquez, José Carlos Escribano Stablé, Adriana Hernández Belmonte, Ana Maestre Peiró, Raquel Martínez Goñi, M.Carmen Pacheco Castellanos, Bernardino Soldan Belda, David Vicente Navarro.

H. G. U. de Castellón. Castellón de la Plana

Jorge Andrés Soler, Marián Bennasar Remolar, Alejandro Cardenal Álvarez, Daniela Díaz Carlotti, María José Esteve Gimeno, Sergio Fabra Juana, Paula García López, María Teresa Guinot Soler, Daniela Palomo de la Sota, Guillem Pascual Castellanos, Ignacio Pérez Catalán, Celia Roig Martí, Paula Rubert Monzó, Javier Ruiz Padilla, Nuria Tornador Gaya, Jorge Usó Blasco.

H. G. U. de Elda. Elda

Carmen Cortés Saavedra, Jennifer Fernández Gómez, Borja González López, María Soledad Hernández Garrido, Ana Isabel López Amorós, Santiago López Gil, Maria de los Reyes Pascual Pérez, Nuria Ramírez Perea, Andrea Torregrosa García

C. A. U. de Salamanca. Salamanca

Gloria María Alonso Claudio, Víctor Barreales Rodríguez, Cristina Carbonell Muñoz, Adela Carpio Pérez, María Victoria Coral Orbes, Daniel Encinas Sánchez, Sandra Inés Revuelta, Miguel Marcos Martín, José Ignacio Martín González, José Ángel Martín Oterino, Leticia Moralejo Alonso, Sonia Peña Balbuena, María Luisa Pérez García, Ana Ramon Prados, Beatriz Rodríguez-Alonso, Ángela Romero Alegría, Maria Sanchez Ledesma, Rosa Juana Tejera Pérez.

Hospital de Palamós. Palamós

Ana Alberich Conesa, Mari Cruz Almendros Rivas, Miquel Hortos Alsina, José Marchena Romero, Anabel Martin-Urda Diez-Canseco.

H. Santa Bárbara. Soria

Marta León Téllez.

H. U. del Sureste. Arganda del Rey

Jon Cabrejas Ugartondo, Ana Belén Mancebo Plaza, Arturo Noguerado Asensio, Bethania Pérez Alves, Natalia Vicente López.

H. Parc Tauli. Sabadell

Francisco Epelde, Isabel Torrente

H. U. Quironsalud Madrid. Pozuelo de Alarcón (Madrid)

Pablo Guisado Vasco, Ana Roda Santacruz, Ana Valverde Muñoz.

Hospital Público de Monforte de Lemos. Monforte de Lemos

José López Castro, Manuel Lorenzo López Reboiro, Cristina Sardiña González.

H. U. Lucus Augusti. Lugo

Raquel Gómez Méndez, Ana Rodríguez Álvarez.

H. Virgen de los Lirios. Alcoy (Alicante)

Ma José Esteban Giner.

Hospital Clínico Universitario de Valladolid. Valladolid

Xjoylin Teresita Egües Torres, Sara Gutiérrez González, Cristina Novoa Fernández, Pablo Tellería Gómez.

Hospital do Salnes. Vilagarcía de Arousa

Vanesa Alende Castro, Ana María Baz Lomba, Ruth Brea Aparicio, Marta Fernández Morales, Jesús Manuel Fernández Villar, María Teresa López Monteagudo, Cristina Pérez García, Lorena Rodríguez Ferreira, Diana Sande Llovo, Maria Begoña Valle Feijoo.

H. de la Axarquía. Vélez- Málaga

Antonio López Ruiz.

H. Virgen del Mar. Madrid

Thamar Capel Astrua, Paola Tatiana Garcia Giraldo, Maria Jesús González Juárez, Victoria Marquez Fernandez, Ada Viviana Romero Echevarry.

H. Comarcal de Blanes. Blanes

Oriol Alonso Gisbert, Mercé Blázquez Llistosella, Pere Comas Casanova, Angels Garcia Flores, Anna Garcia Hinojo, Ana Inés Méndez Martínez, Maria del Carmen Nogales Nieves, Agnés Rivera Austrui, Alberto Zamora Cervantes.

H. U. HM Montepríncipe. Madrid

Jose F. Varona

Hospital Quironsalud A Coruña. A Coruña

Hector Meijide Miguez

## References

[1] Ambrosino I., Barbagelata E., Ortona E., Ruggieri A., Massiah G., Giannico O.V., Politi C., Moretti A.M. (2020). Gender differences in patients with COVID-19: A narrative review. Monaldi Arch. Chest Dis..

[2] Jin J.-M., Bai P., He W., Wu F., Liu X.-F., Han D.-M., Liu S., Yang J.-K. (2020). Gender Differences in Patients With COVID-19: Focus on Severity and Mortality. Front. Public Health.

[3] World Health Organization (2020). WHO Coronavirus Disease (COVID-19) Dashboard. https://covid19.who.int/.

[4] Prasad A., Prasad M. (2020). SARS-CoV-2: The emergence of a viral pathogen causing havoc on human existence. J. Genet..

[5] Klein S.L., Morgan R. (2020). The impact of sex and gender on immunotherapy outcomes. Biol. Sex Differ..

[6] Global Health 50/50: The Sex, Gender and COVID-19 Project. University College of London: c2017–2020. https://globalhealth5050.org/covid19/.

[7] Casas Rojo J.M., Antón Santos J.M., Nuñez-Cortés J.M., Lumbreras C., Ramos Rincón J.M., Roy-Vallejo E., Artero-Mora A., Arnalich-Fernández F., García-Bruñén J.M., Vargas-Núñez J.A. (2020). Clinical characteristics of patients hospitalized with COVID-19 in Spain: Results from the SEMI-COVID-19 Network. Rev. Clin. Esp..

[8] Li L., Huang T., Wang Y., Wang Z., Liang Y., Huang T., Zhang H., Sun W., Wang Y. (2020). COVID-19 patients’ clinical characteristics, discharge rate, and fatality rate of meta-analysis. J. Med. Virol..

[9] Ciminelli G., Garcia-Mandicó S. (2020). COVID-19 in Italy: An Analysis of Death Registry Data. J. Public Health.

[10] European Union Agency European Centre for Disease Prevention and Control. https://www.ecdc.europa.eu/en/covid-19-pandemic.

[11] United Kingdom. Intensive Care National Audit & Researcg Centre. https://www.icnarc.org.

[12] Meng Y., Wu P., Lu W., Liu K., Ma K., Huang L., Cai J., Zhang H., Qin Y., Sun H. (2020). Sex-specific clinical characteristics and prognosis of coronavirus disease-19 infection in Wuhan, China: A retrospective study of 168 severe patients. PLoS Pathog..

[13] Nasiri M.J., Haddadi S., Tahvildari A., Farsi Y., Arbabi M., Hasanzadeh S., Jamshidi P., Murthi M., Mirsaeidi M. (2020). COVID-19 Clinical Characteristics, and Sex-Specific Risk of Mortality: Systematic Review and Meta-Analysis. Front. Med..

[14] Yang T., Barnett R., Jiang S., Yu L., Xian H., Ying J., Zheng W. (2016). Gender balance and its impact on male and female smoking rates in Chinese cities. Soc. Sci. Med..

[15] Palaiodimos L., Kokkinidis D.G., Li W., Karamanis D., Ognibene J., Arora S., Southern W.N., Mantzoros C.S. (2020). Severe obesity, increasing age and male sex are independently associated with worse in-hospital outcomes, and higher in-hospital mortality, in a cohort of patients with COVID-19 in the Bronx, New York. Metabolism.

[16] Borghesi A., Zigliani A., Masciullo R., Golemi S., Maculotti P., Farina D., Maroldi R. (2020). Radiographic severity index in COVID-19 pneumonia: Relationship to age and sex in 783 Italian patients. Radiol. Med..

[17] Mesas A.E., Cavero-Redondo I., Álvarez-Bueno C., Cabrera M.A.S., De Andrade S.M., Sequí-Dominguez I., Martínez-Vizcaíno V. (2020). Predictors of in-hospital COVID-19 mortality: A comprehensive systematic review and meta-analysis exploring differences by age, sex and health conditions. PLoS ONE.

[18] O’Brien J., Du K.Y., Peng C. (2020). Incidence, clinical features, and outcomes of COVID-19 in Canada: Impact of sex and age. J. Ovarian Res..

[19] Qian J., Zhao L., Ye R.-Z., Li X.-J., Liu Y.-L. (2020). Age-dependent Gender Differences in COVID-19 in Mainland China: Comparative Study. Clin. Infect. Dis..

[20] Pijls B.G., Jolani S., Atherley A., Derckx R.T., Dijkstra J.I.R., Franssen G.H.L., Hendriks S., Richters A., Venemans-Jellema A., Zalpuri S. (2021). Demographic risk factors for COVID-19 infection, severity, ICU admission and death: A meta-analysis of 59 studies. BMJ Open.

[21] Słomka A., Kowalewski M., Żekanowska E. (2020). Coronavirus Disease 2019 (COVID–19): A Short Review on Hematological Manifestations. Pathogens.

[22] Zhou Y., Zhang Z., Tian J., Xiong S. (2020). Risk factors associated with disease progression in a cohort of patients infected with the 2019 novel coronavirus. Ann. Palliat. Med..

[23] Fei Z., Ting Y., Ronghui D., Guohui F., Ying L., Zibo L., Xiang J., Wang Y., Song B., Gu X. (2020). Clinical course and risk factors for mortality of adult inpatients with COVID-19 in Wuhan, China: A retrospective cohort study. Lancet.

[24] Kopel J., Perisetti A., Roghani A., Aziz M., Gajendran M., Goyal H. (2020). Racial and Gender-Based Differences in COVID-19. Front. Public Health.

[25] Pradhan A., Olsson P.E. (2020). Sex differences in severity and mortality from COVID-19: Are males more vulnerable?. Biol. Sex Differ..

[26] Sama I., Ravera A., Santema B., Van Goor H., Ter Maaten J., Cleland J., Rienstra M., Friedrich A., Samani N., Ng L. (2020). Circulating plasma concentrations of angiotenis-converting enzyme 2 in men and women with heart failure and effects of renin-angiotensin-aldosterone inhibitors. Eur. Heart J..

[27] Haitao T., Vermunt J., Abeykoon J., Ghamrawi R., Gunaratne M., Jayachandran M., Narang K., Parashuram S., Suvakov S., Garovic V. (2020). COVID-19 and Sex Differences: Mechanisms and Biomarkers. Mayo Clin. Proc..

[28] Sriram K., Insel P.A. (2020). A hypothesis for pathobiology and treatment of COVID-19: The centrality of ACE1/ACE2 imbalance. BJP.

